# 759 Burn Injury Impacts Estrogen-treated Postmenopausal Women

**DOI:** 10.1093/jbcr/irae036.301

**Published:** 2024-04-17

**Authors:** Juquan Song, Georgiy Golovko, Kostiantyn Botnar, Amina E I ayadi, Kathleen L Vincent, Steven E Wolf

**Affiliations:** University of Texas Medical Branch at Galveston, Galveston, TX; University of Texas Medical Branch, GALVESTON, TX; University of Texas Medical Branch, Blocker Burn Unit, Galveston, TX; University of Texas Medical Branch at Galveston, Galveston, TX; University of Texas Medical Branch, GALVESTON, TX; University of Texas Medical Branch, Blocker Burn Unit, Galveston, TX; University of Texas Medical Branch at Galveston, Galveston, TX; University of Texas Medical Branch, GALVESTON, TX; University of Texas Medical Branch, Blocker Burn Unit, Galveston, TX; University of Texas Medical Branch at Galveston, Galveston, TX; University of Texas Medical Branch, GALVESTON, TX; University of Texas Medical Branch, Blocker Burn Unit, Galveston, TX; University of Texas Medical Branch at Galveston, Galveston, TX; University of Texas Medical Branch, GALVESTON, TX; University of Texas Medical Branch, Blocker Burn Unit, Galveston, TX; University of Texas Medical Branch at Galveston, Galveston, TX; University of Texas Medical Branch, GALVESTON, TX; University of Texas Medical Branch, Blocker Burn Unit, Galveston, TX

## Abstract

**Introduction:**

Postmenopausal women are often treated with exogenous female hormones to alleviate mental and physical symptoms. Basic research studies showed the benefit of estrogen treatment on the improvement of cardiac function following burn injury. We posit that women treated with hormone replacement (HRP) fare better following severe burn.

**Methods:**

De-identified patient data were obtained from a global healthcare Research network in March, 2023. Female patients greater than 18 years of age with the diagnoses of menopause were enrolled if they further encountered burn injury within 10 years. Patients with pre-existing gynecologic and chronic liver/heart diseases were excluded. The study population were grouped into those with or without estrogen treatment (ET) for complication examination in both short- and long-term periods . Cohort balancing was performed regarding age, race, and ethnicity using an exact match approach and controlled by the Chi-Square test. Subsequently, the odds ratio (ORs) was computed for these harmonized cohorts. All data manipulation was executed using a custom Python (version 3.10) program in conjunction with Scipy (version 1.11.1) and Pandas (version 2.0.3) libraries.

**Results:**

In the 3-month observational period, women with ET had lower incidence of acute kidney injury and breast cancer [0.475/0.247-0.879, (ORs/95%CI) and 0.362/0.209-0.608]. Other complications were higher in patients with ET including osteoporosis [1.29/1.243-1.596], cerebral infarction [1.735/1.087-2.81], and acute myocardial infarction [3.0/1.776-5.292]. When observation extends to 3 years after injury, patients with ET had similar observations of decreased acute kidney failure [0.236/0.056-0.749], however, increased chronic kidney disease (CKD) [1.203/1.007-1.438], osteoporosis [1.338/11.083-1.654], breast cancer [1.513/1.217-1.885], and acute myocardial infarction (MI) [6.281/1.372-58.484]. All above data were significant with p values < 0.05.

**Conclusions:**

ET in postmenopausal women was associated with decreased acute kidney injury after burn injury, however, also with increased CKD, osteoporosis and MI from 3 months to 3 years after injury. This is not consistent with the ET protective effect seen in rodent burn injury models, therefore, further study on the dose and duration of treatment is warranted as this study in postmenopausal women was primarily low dose, in the range of estrogen or hormone replacement therapy.

**Applicability of Research to Practice:**

Current levels of estrogen provided with Estrogen or Hormone Replacement Therapy do not appear adequate to provide protection that has been seen in preclinical studies, therefore clinical studies to determine dose of estrogen at the time of burn injury should be instituted.

